# Phthisis

**Published:** 1876-03

**Authors:** 


*Phthisis: Its Morbid Anatomy, Etiology, Symptomatic Events, etc.* By AUSTIN FLINT, M. D. Philadelphia: Henry C. Lea. Buffalo: T. Butler & Son.

This volume is the result of observations in six hundred and seventy cases of Phthisis. The author has in this work, as in his previous productions, made use of his own large experience in the treatment of disease ; and hence, what he has to say upon phthisis is the result of conclusions carefully drawn from cases accurately observed. In reference to the morbid anatomy and etiology of phthisis, Dr. Flint gives no new facts. He does not agree with the opinion that it is of inflammatory origin, and in the chapter upon treatment expresses the fear that this view of the disease will lead to the old antiphlogistic methods of treatment. Following the sections upon morbid anatomy and etiology are those upon symptomatic events and complications, fatality and prognosis, treatment, physical signs and diagnosis. In these sections Dr. Flint does not express any views essentially differing from what he has already put upon record.

The chapter upon treatment will be, perhaps, the one to which physicians will turn with the greater interest. He does not consider his experience with the use of arsenic, the hypophosphites, or the pancreatic emulsion of Dr. Dobell, of sufficient extent to form the basis of any conclusions. The author does not think the explanation of the increased number of recoveries over former years can with any correctness be attributed to the employment of cod-liver oil. He is rather inclined to attribute the number of arrested cases to the enlightened views now held concerning alimentation and hygienic treatment, together with a more sparing employment of antiphlogistic measures. Cod-liver oil he regards as a nutriment rather than a drug. Dr. Flint regards alcoholics as signally useful in a certain number of cases, these cases being those in which they can be taken largely without alcoholic excitation or any immediate unpleasant effects. In speaking of the danger of patients becoming addicted to the use of alcoholie stimulants, Dr Flint says that he does not know of a single instance of this nature ; that as a rule patients are glad to discontinue their use when told to do so by their physician. On the subject of physicians being instrumental in making drunkards, he speaks plainly. We quote as follows : " Let me add that I would not be understood to sanction a possible inference that, inasmuch as alcoholics are useful in certain cases in the treatment of phthisis, their use is to be advised by way of prophylaxis, for any who may fancy or affect to fancy themselves in danger of this disease. * * * I do not doubt that the medical profession sometimes receives undeserved censure, because some of those who become addicted to the use of alcoholics, find it convenient to resort, for an excuse, to the falsehood that they were taken nnder the advice of a phpsician."

Dr. Flint makes some valuable suggestions concerning the value of the change of climate in the treatment of phthisis. We have not the space to quote some portions of his remarks upon climate to which we would like to refer. We can simply say that what he says upon the subject seems eminently well weighed and judicious.

The work shows a large amount of patient labor and comparison of caess, and while it presents no new facts, will amply repay a careful study.

